# Progress in the Production of Virus-Like Particles for Vaccination against Hepatitis E Virus

**DOI:** 10.3390/v12080826

**Published:** 2020-07-30

**Authors:** Milena Mazalovska, J. Calvin Kouokam

**Affiliations:** 1 Department of Pharmacology and Toxicology, University of Louisville School of Medicine, Louisville, KY 40202, USA; milena.mazalovska@louisville.edu; 2 Center for Predictive Medicine, University of Louisville School of Medicine, Louisville, KY 40202, USA; 3 James Graham Brown Cancer Center, University of Louisville School of Medicine, Louisville, KY 40202, USA

**Keywords:** Hepatitis E virus, ORF2 capsid protein, HEV VLPs, vaccines

## Abstract

Hepatitis E virus (HEV), a pathogen that causes acute viral hepatitis, is a small icosahedral, quasi-enveloped, positive ssRNA virus. Its genome has three open reading frames (ORFs), with *ORF1* and *ORF3* encoding for nonstructural and regulatory proteins, respectively, while *ORF2* is translated into the structural, capsid protein. *ORF2* is most widely used for vaccine development in viral hepatitis. Hepatitis E virus-like particles (VLPs) are potential vaccine candidates against HEV infection. VLPs are composed of capsid subunits mimicking the natural configuration of the native virus but lack the genetic material needed for replication. As a result, VLPs are unable to replicate and cause disease, constituting safe vaccine platforms. Currently, the recombinant VLP-based vaccine Hecolin^®^ against HEV is only licensed in China. Herein, systematic information about the expression of various HEV *ORF2* sequences and their ability to form VLPs in different systems is provided.

## 1. Introduction

Hepatitis E virus (HEV) is an enterically transmitted pathogen and a major cause of acute hepatitis in many developing countries within Africa and Asia [1]. Approximately one third of the world population live in areas in which HEV is endemic and thus are at risk of infection [2]. Unlike other viruses causing hepatitis, HEV-related disease is a zoonotic infection with pigs, wild boars and certain other species such as deer and rabbits being considered as reservoirs for the virus [3,4]. Although the fatality rate during epidemics is low, i.e., between 0.2–5% [5], the mortality rate in pregnant women is as high as 25%, possibly due to altered hormone status and decreased immunity [6,7,8]. Even though HEV infection is considered self-limiting or asymptomatic in healthy individuals, it can lead to severe disease in patients with preexisting liver conditions, with high morbidity and mortality [9,10]. Chronic infection could develop in immunocompromised patients such as organ transplant recipients [11], individuals administered immunosuppressants [12], patients on chemotherapy for hematological malignancies [13], HIV-infected patients [14] and cases of superinfection with other hepatitis viruses [15]. In 10% of chronically infected patients, HEV leads to rapid progression to liver cirrhosis in less than 3 years [16]. In addition, it has become evident in recent years that HEV infections can be associated with neurological manifestations [17,18], renal aliments [19], hematological disorders [20] and acute pancreatitis [21]. Furthermore, recent data indicate a link between HEV infection and progression to hepatocellular carcinoma in patients infected with hepatitis B virus (HBV) or hepatitis C virus (HCV) [22,23]. Atsama et al. [22] reported significantly higher prevalence of anti-HEV IgG in hepatocellular carcinoma (HCC) patients infected with either HBV or HCV compared with HBV/HCV-infected patients with chronic liver disease but not suffering from HCC [22]. This finding suggests that infection with HEV could worsen liver inflammation and increase the severity of other infections. Another study also reported that HEV superinfection accelerates the progression of chronic HBV infection and increases 1-year mortality [23]. 

Traditional approaches for the development of an HEV vaccine have been ruled out because the manufacturing of either live attenuated or inactivated vaccine would be impossible due to the complexity and low yield of viral culture. Even though culturing the virus has been difficult in the past, a few strains have been adapted to cell culture, leading to a better understanding of the HEV life cycle [24].

Presently, significant progress has been made in the development of HEV vaccines based on the ORF2 capsid protein as either a subunit or virus-like particle (VLP) [25]. VPLs represent one of the most attractive systems for vaccine development due to their safety, immunogenic properties and ease of production [26]. VLPs are generated from one or more viral capsid proteins that self-assemble into high-molecular-weight structures that resemble the native virions but lack the viral genome [27]. As a result, VLPs are replication- and infection-incompetent, making them a safe alternative to attenuated or inactivated viruses in vaccine development. Since they are structurally similar to the native virus, they can induce stronger B and T cell responses than traditional small subunit vaccines [28]. Additionally, VLPs can be better taken up by professional antigen-presenting cells (APCs) as exogenous and endogenous antigens for processing and presentation by MHC class II and I molecules, respectively. Cross-presentation by MHC class molecules activates CD4+ and CD8+ T cells that elicit specific cytotoxic T lymphocyte (CTL) responses resulting in infection control [29]. Furthermore, VLPs can be assembled not only from proteins from a single virus, but also from proteins of distinct viruses or various other pathogens, e.g., bacteria and protozoa [30]. To date, several VLPs have been produced for protection against infectious diseases in prokaryotic or eukaryotic expression systems [31], and in some cases assembled in cell-free conditions [32]. Some of these products have been licensed, including Engerix^®^ (Hepatitis B virus) [33], Cervarix^®^ (human papilloma virus) [34], Recombivax HB^®^ (HBV) [35] and Gardasil^®^ (HPV) [36], while others are still under pre-clinical and clinical evaluation [37,38]. This review summarizes the basic information about HEV genome organization, the expression of ORF2 capsid protein in several expression systems and current progress in developing VLP-based HEV vaccines. In addition, we described in detail all recombinantly expressed HEV sequences discovered in humans and animal species as well as the amino acid sequences required for VLP formation with different levels of success. 

## 2. Hepatitis E Genome Organization

Previously known as non-A non B hepatitis, HEV is currently classified in the *Hepeviridae* family with the two genera *Orthohepeviruses* and *Pischihepeviruses* [39]. The *Orthohepevirus* A genus includes genotypes 1 and 2 isolated from humans, genotypes 3 and 4 from both humans and animals, the newly proposed genotypes 5 and 6 from wild boars and genotype 7 from dromedary camels [40,41]. 

**Figure 1:**
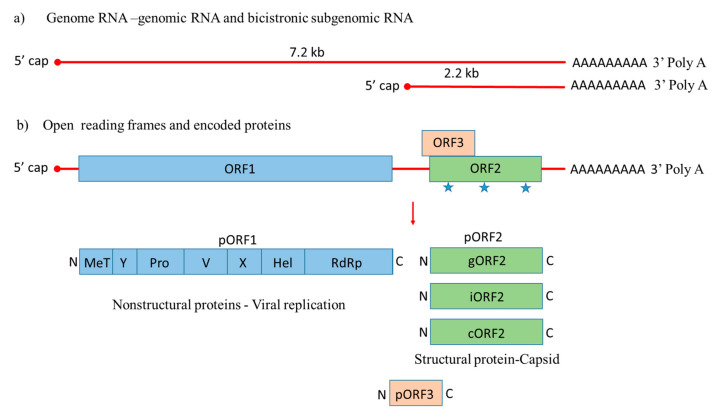
Genome organization of Hepatitis E virus. (**a**) Hepatitis E-Virus (HEV) genome generates the full-length genomic RNA and subgenomic RNA with 5′ cap, 3′ Poly A tail, 5′ UTR and 3′ UTR. (**b**) The genomic RNA has three open reading frames: *ORF1*, *ORF2*, and *ORF3*. *ORF1* encodes the nonstructural proteins for viral replication; *ORF2* is translated into the capsid protein with three potential glycosylation sites (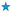), with a small multifunctional protein encoded by *ORF3*. Three different capsid proteins have been discovered in vitro during infection, i.e., gORF2-glycosylated, iORF2-infectious and cORF2-cleaved ORF2.

HEV is a quasi-enveloped, icosahedral, single-stranded positive-sense RNA virus that was molecularly characterized for the first time in 1990 [42]. Its genome is around 7.2 kb with features of a eukaryotic mRNA, including a 5′ cap and 3′ poly A tail, 5′ and 3′ untranslated regions (UTRs), and three open reading frames, including *ORF1*, *ORF2*, and *ORF3* [43]. During HEV genome replication two viral RNA species are generated, i.e., the full-length genomic RNA and a subgenomic RNA [44]. The subgenomic RNA allows the expression of *ORF2* and *ORF3* (Figure 1).

*ORF1* encodes nonstructural proteins involved in viral replication [45,46]. A small multifunctional 13 kDa protein is expressed from *ORF3*, which facilitates HEV transport throughout the cell and acts as viroporin for the release of the infectious virus from the host cell [47,48]. *ORF2* encodes the 72 kDa capsid protein comprising 660 amino acids that contains a hydrophobic stretch of 14–34 amino acids at the N-terminus, which functions as a signal sequence for its secretion [49]. ORF2 is involved in virion assembly, attachment to the host cell and immunogenicity [50,51,52]. Additionally, the capsid protein has three potential glycosylation sites (Asn 132, 310 and 562) [53]. 

Native HEV particles are round non-enveloped with spikes covering the surface [54,55]. It is considered that 180 copies of the ORF2 protein form the HEV virion giving it T = 3 icosahedral symmetry [56]. Recently, a few strains have been adapted for replication in cell culture, providing novel insights into the HEV cycle. Even though HEV particles present in the bile and feces are non-enveloped, it was demonstrated that in patient serum and cell cultures, HEV particles are partially associated with lipids and the ORF3 protein [57]. Moreover, recent studies have identified different forms of ORF2 in cultured cells. Large ORF2 protein amounts are released from HEV-infected cells in vitro and found in serum from HEV-infected patients. This secreted protein (ORF2s) was shown to be glycosylated form of the capsid protein that is not associated with the HEV virion. The other intracellular protein (ORF2c), a translation product of the same gene starting with the second AUG codon, is involved in HEV assembly [58]. Montpellier et al. reported iORF2 (infectious), gORF2 (glycosylated) and additional ORF2 truncated protein (ORF2c) are not involved in virion assembly using another genotype and cell culture for replication [59]. 

Great efforts have been made towards understanding the HEV life cycle in recent years by developing cellular systems and infectious HEV clones [60]. Polarized cell models have been developed to closely mimic in vivo infection with HEV, which are highly permissive to infection, making them a good tool for molecular studies of the HEV cycle. For example, human hepatoma-derived HepaRG and porcine hepatocyte-like PICM-19 cell lines have been shown to support HEV replication, and are useful for studying virus–host interactions and species barrier crossing, especially since HEV infection is a zoonosis in developed countries [61]. Capelli et al. [62,63] showed that different HEV genotypes release more than 90% of the virus from the apical membrane after infecting polarized human hepatocellular carcinoma HepG2/C3A cells, suggesting the main route of release for infectious virions [62,63]. In recent years, the key steps of HEV’s natural infectious cycle in vivo have been confirmed by employing polarized human stem-cell-derived, hepatocyte-like cells (HLCs). Infection of these cells with HEV results in the secretion of two different progeny particle types, including quasi-enveloped particles from the basolateral membrane and naked highly infectious virions from the apical membrane [64]. These findings provide novel insights into the HEV infectious cycle. The release of HEV particles basolaterally could spread the infection in the host and lead to extrahepatic manifestations [65].

## 3. VLP-Based Vaccines for HEV Prevention 

Improving the sanitary conditions in endemic areas would significantly curb HEV infection incidence; however, vaccination is also needed for protection. In non-enveloped viruses, the capsid not only protects the viral nucleic acid, but is also involved in cell receptor binding, virus internalization, and genome release into the cytoplasm. Prevention of HEV infection by vaccination relies on the capsid protein as it is highly immunogenic and elicits effective virus-neutralizing antibodies [25,66]. Previous studies suggest that the neutralization epitopes are located in the C-terminal region of the capsid protein [55,67], with residues 458–607 of ORF2 being the shortest neutralization fragment [68]. By contrast, proteins translated from *ORF1* are immunogenic but do not confer protection since they are not part of the virion. Furthermore, antibodies raised against the small ORF3 protein are produced during infection; however, they are short-lived and have no neutralizing capability [69]. As a result, research is currently focused on expressing the capsid protein for the development of prophylactic vaccines. To date, three vaccine candidates have been evaluated in clinical trials. Two of them are produced in *E. coli* as VLPs, including p179 and p239 [70]. The third one, a 56 kDa recombinant protein produced in insect cells, has undergone phase II clinical trials [71]. To date, several different systems have been used to express the HEV capsid protein, including *E. coli* [72], insect cells [73], mammalian cells [49] and plants [74]. However, *E. coli* and the baculovirus-insect cell system are considered the most effective systems for producing HEV VLPs [70,73,75].

### 3.1. Expression of HEV ORF2 in E. Coli

Several HEV ORF2 proteins with different lengths have been expressed and purified in *E. coli* in order to determine their particle-forming properties (Table 1). The shortest protein, termed E2s (459–606 aa), represents the minimal requirement for the formation of dimers in solution, contains the neutralizing site, and is considered to be necessary for virus–host interaction [76,77]. Another fragment, pE2, comprising amino acids 394–606 of HEV-1 (Chinese strain) ORF2 also forms dimers upon expression in *E. coli*. It was shown that pE2 is recognized strongly by HEV reactive human sera in its dimeric rather than monomeric form, suggesting that the dimer could mimic the structural features of the virus capsid [72]. Not surprisingly, immunization of macaques with the peptide triggered a strong antibody response and prevented experimental HEV infection of the animals [78]. Moreover, pretreatment with two monoclonal antibodies raised against pE2, diminishes HEV infectivity in rhesus monkeys, providing further support that the pE2 dimer models the 3D features of HEV’s native capsid [79]. Adding 26 amino acids toward the N-terminus of the pE2 peptide results in higher-order assembly structures beyond dimerization. Expressed in *E. coli*, p239 (HEV 239) forms VLPs with a diameter of 23 nm. These VLPs (HEV 239) are highly immunogenic in both rhesus macaques and mice [75,80]. Even though HEV 239 and pE2 have similar antigenic activities, HEV 239 appears to be 200 times more immunogenic compared with pE2. Unlike pE2, HEV 239 can induce vigorous antigen-specific T-cell response in mice [81]. Generally, the formation of VLPs is considered key for immune recognition and response, and HEV 239 has been further studied as a potential vaccine candidate [82]. Since the latter showed immunogenicity in preclinical experiments, it was approved for use in human trials. HEV 239 VLPs were shown to be safe and immunogenic in humans in a phase II clinical trial of seronegative patients [83]. In addition, more than 110,000 individuals participated in a phase III trial of the vaccine candidate HEV 239 that showed 100% efficacy over the 12 month period after three immunizations [84]. To date, the HEV 239 (Hecolin^®^) vaccine has been licensed for use only in China [70,85]. Recent research showed that the HEV vaccine could provide long-term protection, i.e., up to 4.5 years, with 86.6% efficacy [86]. In order to be recommended for global use, the HEV239 vaccine must be further investigated for safety and protection in various risk groups. Currently, several studies have shown that the p239-based vaccine is well tolerated in the elderly population (>65 years old) [87] and hepatitis B surface antigen (HbsAg)-positive adults [88]. In addition, two clinical trials evaluating the HEV239 vaccine are ongoing, including: a phase I safety study in the USA (NCT03827395) and a phase IV trial in Bangladesh (NCT02759991) evaluating its safety and efficacy in pregnant women, who are at higher risk of acute liver failure and elevated neonatal mortality and morbidity [89]. The most recent VLP vaccine, termed p179 (439–617 aa of ORF2 protein), derived from HEV genotype 4, has been developed and assessed in a phase I clinical trial in China [90]. The p179 vaccine candidate was found to be safe and well tolerated among the included participants. The p179 and p239 vaccines showed a difference in immunogenicity due to possible genotype-specific neutralization epitopes, which raises questions about the effectiveness of a vaccine towards different genotypes and the need to develop a vaccine with broader efficacy [91]. Even though p239 is the shortest sequence necessary for assembly in VLPs, it was also shown that a longer sequence of ORF2 112–606 aa (p495) can self-assemble into VLPs after expression in *E. coli*. The difference between both peptides is that p495 is capable of self-assembly in vitro [92].

### 3.2. Expression of HEV VLPs in the Baculovirus-Insect Cell System

The baculovirus expression system has been used extensively for the production of recombinant proteins [100,101,102]. This system is an attractive platform for protein expression for several reasons, including a rapid growth rate, easy scalability, and eukaryotic posttranslational modifications of the expressed proteins [103]. The first commercially available vaccine (Cervarix^®^) expressed in this system was based on HPV type 18 and 16 L1 VLPs, and used to prevent human papillomavirus infections [104,105]. Additionally, many other VLPs have been produced in the baculovirus-insect cell system such as Influenza A [106], Norwalk virus [107], Bluetongue virus [108] and Chikungunya virus VLPs [109]. 

The baculovirus-insect cell system is the most extensively applied and successful system for the expression of HEV as VLPs to date. Initially, expression of the whole ORF2 protein in the Sf9 cell line yielded several proteins with different molecular weights (72, 63, 56 and 53 kDa), none of which self-assembled into VLPs [73,110]. These proteins were immunoreactive and characterized as by-products of the whole protein undergoing a series of truncations at the N-and/or C-terminus after expression in Sf9 cells. Among these, the 56 kDa protein was the only protein evaluated as a potential vaccine in a phase II clinical trial in Nepal [71]. This protein was shown to be highly immunogenic in cynomolgus monkeys, and after two administrated doses the animals were protected against virulent HEV [111,112]. Despite promising results, this vaccine candidate did not undergo further clinical development, possibly due to limited commercial potential [113]. The first report of the assembly of HEV VLPs in insect cells dated 1997 [73]. After expressing the whole HEV *ORF2* sequence, the three major proteins 72, 58 and 50 kDa were found in two different insect cell lines, including Tn5 and Sf9 cells. Despite their immunoreactivity, these proteins were cell-associated and did not form VLPs. However, expression of ORF2 with a truncation of the N-terminal 111 residues in Tn5 cells produced two proteins of 58 and 50 kDa, respectively, with the latter found in the culture medium as VLPs of 23–24 nm (Table 1) [73]. It was concluded that both the N-terminal truncation and the cell line used for expression are important for VLP formation. Additionally, the 50 kDa (C- and N-end truncated ORF2) protein expressed in Sf9 cells could assemble into VLPs [76]. Li et al. with a series of truncations of the capsid protein, determined that the core structure of the ORF2 protein that can form VLPs is in the 126–601 aa range [75]. The HEV capsid protein can self-assemble into either a small VLP composed of 60 copies of a truncated ORF2 (112–608 aa) with T = 1 symmetry [67] or a large VLP made up of 180 capsid subunits (14–608 aa) with T = 3 symmetry [56]. Since large VLPs have different symmetry than the small ones, the additional amino acids (14–112 aa) at the N-terminus seem to play a role in the efficient formation of T = 3 symmetry capsids and the ability to encapsulate RNA [114]. 

#### 3.2.1. Formation of VLPs from Animal HEV Sequences in the Baculovirus-Insect Cell System

With the discovery of new HEV strains in many animals such as rats [96], camels [98], wild boars [99], and ferrets [115], HEV has the potential to cause a serious veterinary problem, hence the need for research into animal-specific strains of HEV. Li et al. expressed the rat sequence of HEV with the same genome organization as genotype 1–4 in two different cell types [96,116]. The only sequence that could form VLPs in the Tn5 cell line was the 110–660 aa peptide, corresponding to the 112–660 sequence of genotype 1, which produced two proteins, p58 and p53 [96]. Electron microscopy assessment of purified p53 revealed two types of VLPs, with diameters of 24 and 35 nm, respectively (Table 1). The morphology of the particles was similar to that of other HEV VLPs purified previously, and they were apparently empty [96]. The same group also expressed the HEV *ORF2* sequence, which was firstly discovered in ferrets in the Netherlands [115]. Alignment showed that amino acids 19–113 in the ferret ORF2 sequence correspond to amino acids 14–112 in genotypes 1–4. Even though VLPs were still generated after the deletion of amino acid 13 or 111 from the N-terminus in genotypes 1–4, deletion of the corresponding sequence in the ferret ORF2 sequence abolished the assembly of VLPs [97]. Only the sequence with truncations at both termini (112N/47C) could [97]. With the discovery of a novel HEV in camels (DcHEV), two of these sequences have been expressed to assess their antigenicity and pathogenicity. The authors determined that expressed 13N truncated proteins could form large VLPs and package RNA after the deletion of an additional 50 amino acids at the C-terminus. The expression of 111N truncated proteins resulted in small VLPs only in one of the sequences. These two proteins had differences in only two amino acids, with one (a methionine residue at position 358) being uncommon compared to other ORF2 sequences, demonstrating that even small changes to the primary sequence can affect VLP formation [98]. Two other sequences isolated from wild boars pertaining to genotypes 5 and 6 have also been expressed. The expression of the 111N ORF2 truncation yielded two proteins, including the 58 and 53 kDa peptides, of which the latter could self-assemble in VLPs with a diameter of 24 nm, similar to that of other HEV VLPs produced in this system [99]. On the other hand, the 13N truncation of both ORF2 sequences yielded proteins with different molecular weights, i.e., 71, 64, 53 and 40 kDa. Only the 64 kDa protein could form VLPs with a diameter of 35 nm resembling the native viral particle. None of the human and animal ORF2 sequences could form VLPs after expression of the whole ORF2 protein; for VLP formation, N-terminal and/or C-terminal truncations were needed for assembly in insect cells. More research is needed to comprehensively determine what sequences are necessary for VLP formation.

#### 3.2.2. HEV VLPs as a Platform for Foreign Epitopes 

Since HEV is an enterically transmitted virus and spreads through drinking of contaminated water, it is a good candidate for developing an oral vaccine. The benefits of oral over parenteral immunization include cost reduction, better adherence and easy delivery. Therefore, rHEV VLPs produced in insect cells were tested to determine whether they could be used for oral immunization in mice and cynomolgus monkeys. The results showed that rHEV VLPs are highly immunogenic and trigger the immune response without adjuvant application in both animal models [117,118]. Additionally, HEV VLPs can be used as a platform to present foreign epitopes. Niikura et al. showed that expressing the HEV capsid with a B cell epitope tag at the C-end of the protein does not disrupt VLP formation. Additionally, the chimeric VLPs induced an antibody response to both the tagged and HEV-VLPs [119]. The insertion of an epitope, such as the p18 peptide derived from the V3 loop of HIV-1 gp120, at the antibody-binding site in the P domain still allowed the formation of VLPs, which do not react to anti-HEV antibodies, suggesting that chimeric HEV VLPs could escape pre-existing immunity and constitute a platform for the presentation of foreign epitopes [119]. Furthermore, Shima et al. showed that HEV VLPs might be a vehicle for a multivalent mucosal vaccine by co-expressing capsid proteins with different tags and/or neutralizing epitopes from the Japanese encephalitis virus, allowing the formation of chimeric HEV VLPs that display divalent or trivalent foreign epitopes on its surface [120]. These examples show the extensive opportunities of HEV VLPs for the display of heterologous antigens.

### 3.3. Expression of ORF2 as VLPs in Plants

Since the first plant-derived recombinant protein, human serum albumin, was produced in transgenic tobacco in 1990 [121], plants have been used to successfully express a variety of other therapeutic proteins, blood components, cytokines, hormones, growth factors, vaccines and antibodies [122]. This resulted in federal approval (US Department of Agriculture Center for Veterinary Biologics) in 2006 of the first plant-made vaccine against Newcastle disease in poultry developed by Dow AgroSciences LLC (Indianapolis, India). Plants offer several advantages compared with other recombinant protein expression systems. These include the presence of eukaryotic post-translational modification machinery, simple and low-cost scale-up for manufacturing, and the inability to transmit human pathogens through the manufacturing medium [123,124]. Another advantage of using plants for the production of biopharmaceuticals is that products expressed in edible plant organs could be administered directly as oral vaccines in the form of unprocessed plant materials [125]. 

The first study expressing truncated ORF2 in plants was performed in transgenic tomatoes, which could enable the production of an oral vaccine against HEV with easy administration and low-cost production (Table 1). The pE2 region corresponding to residues 394–604 aa was introduced into Agrobacterium tumefaciens, and ELISA showed that the transgenic plants produced the truncated ORF2 protein, which had normal immunoactivity [74]. Unfortunately, in transgenic tomatoes the truncated ORF2 protein does not accumulate to a high level (48–61 ng/g FW). In an attempt to improve pE2 yield, its sequence was transformed into the plastid genome. Transplastomic plants have been shown to express certain proteins at high levels due to the presence of multiple copies of the plastid genome [126]. Transplastomic tobacco (*Nicotiana tabacum* cv. SR1) plants were obtained by inserting a vector for chloroplast-targeting containing the pE2 peptide (394–607 aa) into the plastid genome using biolistic particle bombardment of leaf pieces. The transformed plants expressed pE2 at higher levels (13.27 μg/g FW) compared with transgenic tomatoes, and the pE2 peptide was antigenic in mice [93]. Maloney et al. engineered a transgenic potato line expressing *ORF2* in order to develop an oral vaccine [94]. Two different lengths of the capsid protein, including pHEV101 (111N) and pHEV110 (111N/52C truncation), were used, that had previously been shown to form VLPs in insect cells [73]. Western blot analysis showed that expressing both genes in transgenic potatoes results in proteins with a size of 54 kDa (the correct size of pHEV101 but not for pHEV110) and some lower MW degradation products. It was proposed that since the two truncations produce the same size protein, the 52 C-terminal amino acids in pHEV110 must be removed by the plant itself. Oral immunization of mice with potatoes expressing the capsid protein was unsuccessful in producing detectible antibody response, mostly because the expressed proteins do not assemble into VLPs [94]. Additionally, transient expression of truncated ORF2 (110–610 aa) sequence from swine HEV-3 with the highly efficient vector pEAQ-HT was performed in Nicotiana benthamiana plants for the first time [127,128]. The truncated protein was purified in high amounts of up to 100 mg/kg FWT, and could be used as a diagnostic antigen [95]. Attempts were also made to produce HEV VLPs and chimeric M2-HEV VLPs using varying lengths of ORF2 [129]. The immunogenicity and VLP assembly properties of the transiently expressed ORF2 in plants remain to be determined.

## 4. Discussion

It is estimated that 20.1 million infections, 70,000 deaths and 3000 stillbirths result from HEV genotype 1 and 2 infections in Africa and Asia [130]. Moreover, the possible zoonotic spread of genotypes 3 and 4 through direct contact with or consumption of contaminated food products additionally raises health concerns over the zoonotic risk of Hepatitis E infection [131,132]. Although HEV is a self-limiting disease in immunocompromised patients, such as transplant patients and HIV-infected individuals, it leads to chronic infection [133]. Acute and chronic HEV infections, compounded with the zoonotic spread of the virus, impose even a greater burden on healthcare systems globally. More than ever, an effective vaccine against HEV is needed. 

Since the discovery of HEV, knowledge about the virus, its replication cycle, virion structure and usage for vaccine development or diagnostics has been limited by its inability to grow efficiently in tissue culture. The most current data about HEV biology are based on RNA replicons and transient transfection in cell cultures. Hepatitis E infection, similar to many other infectious diseases, is preventable by vaccination. The difficult HEV propagation has also hindered the cost-effective and extensive production of VLPs as vaccine candidates. The ORF2 is the most widely used protein for HEV vaccine development because it encodes a single capsid protein involved in genome encapsidation, attachment to host cells, and immune response induction [50,51,52].

Due to the lack of propagation systems for the virus, most efforts in developing HEV VLP vaccines have focused on recombinant ORF2 capsid protein production in several expression systems. To date, it has been demonstrated that the protein most effectively self-assembles into VLPs in the baculovirus-insect cell system [73]. However, the disparate results upon the expression of different genotype sequences in two different insect cell lines compounded with varying lengths of the capsid protein forming VLPs indicates that more research is needed to determine the factors affecting assembly. Interestingly, with the development of an HEV strain that can replicate in cell culture, we are closer to understanding the role of ORF2 in HEV infection and pathogenesis. Recent studies have shown that, during infection, multiple forms of the ORF2 protein are produced, explaining previously reported results [58,59]. That most of the produced and secreted ORF2 is not associated with the virion could potentially explain the lack of VLPs upon expression of the whole protein in insect cells. The presence of the capsid protein in the serum of infected patients reveals a new role for ORF2 in HEV infection [58]. It was reported that the sequence of ORF2 involved in the virion formation starts from the second internal codon 14 aa from the starting codon [58]. This could potentially explain why only the truncated ORF2 could form VLPs upon expression in insect cell lines. Another group expressing ORF2 from genotype 4 reported three different ORF2 sequences, of which only one is involved in virion formation [59]. Whether these differences are based on the different cell lines used or distinct genotypes deserves further investigation. In addition, HEV replication and infection in humans and animals is not completely defined.

## 5. Conclusions and Future Directions

Overall, HEV infection remains a global challenge, with Africa and Asia greatly affected. In addition, the virus can spread to humans from animals. An increasing number of HEV sequences have been detected in various animal species, some of which have been confirmed as natural reservoirs for HEV and sources of zoonotic infection. This raises concerns about the potential risk of cross-species infection and zoonotic transmission. Even though HEV infection is mostly asymptomatic, immunocompromised individuals have a considerable risk of developing chronic infection. Therefore, there is an urgent need for HEV vaccines for global use, especially in outbreak regions and high-risk groups. Currently, studies developing HEV VLP vaccines have mostly focused on producing recombinant ORF2 capsid protein in various expression systems. To date, the protein is known to most effectively self-assemble into VLPs in the baculovirus-insect cell system and *E. coli*. However, the sole VLP-based vaccine Hecolin^®^ for HEV prevention is only licensed in China, showing 100% efficacy. Additional studies are under way to assess the safety and efficacy of HEV239 in high-risk groups for potential global distribution, as recommended by the WHO, However, this vaccine is derived from a single genotype; with newly identified HEV isolates from animal species that infect humans, it remains unknown whether it could provide immunity against other genotypes. Therefore, further investigation is needed to address its potential pitfalls and/or to design efficient chimeric VLPs with broad-cross protection.

## Figures and Tables

**Table 1:** Expressed HEV ORF2 protein and VLPs formation in different systems.

Organism	HEV ORF2	Molecular Weight	VLPs	T Number	RNA	Remarks	Ref:
Bacteria E. coli	E2s (459–606 aa)	16 kDa	HEV 239 (p239) 20–30 nm	n/a	n/a	Hecolin^®^ licensed vaccine only in China	[77]
	E2 (394–606 aa)	23 kDa					[72]
	p239 (368–606 aa)	30 kDa					[80]
	P179 (439–617 aa)	20 kDa					[90]
	p495 (112–606 aa)	53 kDa					[92]
Transgenic tomato plants	E2 (394–606 aa)	23 kDa	Limited assembly of VLPs	n/a	n/a	No success so far in production of VLPs in plants	[74]
Tobacco plastids	E2 (394–606 aa)	23 kDa					[93]
Transgenic plants	112–660 aa 112–608 aa	54 kDa					[94]
*Nicotiana benthamiana*	110–610 aa	56 kDa					[95]
Baculovirus-Insect cells system	112–660 aa (genotype 1)	58, 50 kDa	50 kDa VLPs ~23 nm	T = 1	No	Expression of the whole ORF2 does not form VLPs	[73]
Tn5 cell line	Rat 110–660 aa	58, 53 kDa	53 kDa VLPs ~24, 35 nm	T = 1; T = 3	No		[96]
	Ferret 112–613 aa	53 kDa	VLPs ~24 nm	T = 1	No		[97]
	Camel 13–610 aa	70, 64, 53, 40 kDa;	64 kDa VLPs ~35 nm	T = 3	Yes	For VLPs formation N-terminal truncation is needed.	[98]
	111–610 aa	58 и 53 kDa	53 kDa VLPs ~24 nm	T = 1	No		
	Wild boar 112–660aa	58 and 53 kDa;	53 kDa VLPs ~24 nm	T = 1	No		[99]
	13–660 aa	71, 64, 53, 40 kDa	64 kDa VLPs ~35 nm	T = 3	Yes		
